# Strigolactones spatially influence lateral root development through the cytokinin signaling network

**DOI:** 10.1093/jxb/erv478

**Published:** 2015-10-31

**Authors:** Lingxiang Jiang, Cedrick Matthys, Belen Marquez-Garcia, Carolien De Cuyper, Lien Smet, Annick De Keyser, François-Didier Boyer, Tom Beeckman, Stephen Depuydt, Sofie Goormachtig

**Affiliations:** ^1^Department of Plant Systems Biology, VIB, 9052 Gent, Belgium; ^2^Department of Plant Biotechnology and Bioinformatics, Ghent University, 9052 Gent, Belgium; ^3^Institut Jean-Pierre Bourgin, Unité Mixte de Recherche 1318, Institut National de la Recherche Agronomique-AgroParisTech, 78026 Versailles Cedex, France; ^4^Centre de Recherche de Gif, Institut de Chimie des Substances Naturelles, Unité Propre de Recherche 2301, Centre National de la Recherche Scientifique, 91198 Gif‐sur‐Yvette, France; ^5^Ghent University Global Campus, Incheon 406–840, Korea

**Keywords:** *Arabidopsis thaliana*, cytokinin signaling, lateral root development, polar auxin transport, strigolactones.

## Abstract

Strigolactones are important rhizosphere signals that act as phytohormones and have multiple functions, including modulation of lateral root (LR) development. Here, we show that treatment with the strigolactone analog GR24 did not affect LR initiation, but negatively influenced LR priming and emergence, the latter especially near the root–shoot junction. The cytokinin module *ARABIDOPSIS HISTIDINE KINASE3* (*AHK3*)/*ARABIDOPSIS RESPONSE REGULATOR1* (*ARR1*)/*ARR12* was found to interact with the GR24-dependent reduction in LR development, because mutants in this pathway rendered LR development insensitive to GR24. Additionally, pharmacological analyses, mutant analyses, and gene expression analyses indicated that the affected polar auxin transport stream in mutants of the *AHK3/ARR1/ARR12* module could be the underlying cause. Altogether, the data reveal that the GR24 effect on LR development depends on the hormonal landscape that results from the intimate connection with auxins and cytokinins, two main players in LR development.

## Highlight

Strigolactones monitor lateral root development in a spatiotemporal manner by an interplay with cytokinin.

## Introduction

Strigolactones (SLs) are phytohormones that affect lateral branching of the shoot (Gomez-Roldan *et al.*, 2008; Umehara *et al.*, 2008) and many other processes, such as photomorphogenesis, drought tolerance, leaf senescence, and secondary growth, among others (Woo *et al.*, 2001; Snowden *et al.*, 2005; Shen *et al.*, 2007, 2012; Tsuchiya *et al.*, 2010; Agusti *et al.*, 2011; Bu *et al.*, 2014). In the rhizosphere, SLs influence interactions of the host plant with neighboring organisms, such as root-parasitic plants, mycorrrhizal fungi, and rhizobia (for review, see Xie *et al.*, 2010; Rasmussen *et al.*, 2013a). The root system architecture itself is also affected by SLs, because SLs influence adventitious root development, main root growth, root hair development, and lateral root (LR) development (Kapulnik *et al.*, 2011a, 2011b; Ruyter-Spira *et al.*, 2011; Mayzlish-Gati *et al.*, 2012; Rasmussen *et al.*, 2012, 2013a; Sun *et al.*, 2014). The ontogenesis of LRs consists of several successive steps that are highly regulated (reviewed by Péret *et al.*, 2009). The first step is priming of the LR that occurs in the xylem pole pericycle (XPP) cells in the basal meristem zone of the root tip. These primed XPP cells, also designated prebranch sites, have acquired the developmental program to become an LR. As the root grows, the primed XPP cells enter the elongation zone, where they undergo asymmetric cell division, a process designated LR initiation. Through further well controlled division patterns, an LR primordium will be formed that will ultimately develop into a typical dome-shaped primordium that will pierce through the main root and will form an emerged LR.

Regarding LR development, addition of the SL analog GR24 was found to reduce the LR density (LRD), because of a diminished LR initiation and LR outgrowth (Koltai *et al.*, 2010; Kapulnik *et al.*, 2011b; Ruyter-Spira *et al.*, 2011). In *Arabidopsis thaliana*, mutants in the F-box protein MORE AXILLARY GROWTH2 (MAX2) are perturbed in SL perception and display higher LRDs than the wild-type (WT) plants (Kapulnik *et al.*, 2011b; Kohlen *et al.*, 2011; Ruyter-Spira *et al.*, 2011). When MAX2 function was restored specifically in the root endodermis of *max2* mutants, their insensitivity could be partially complemented (Koren *et al.*, 2013). SLs are perceived by an α/β-hydrolase, DWARF14 (D14), that binds and hydrolyzes SLs and plays a central role in downstream signaling activation (Hamiaux *et al.*, 2012; Zhao *et al.*, 2013). In petunia (*Petunia hybrida*) and rice (*Oryza sativa*), D14 interacts with MAX2/D3, a nuclear-localized F-box protein that participates in the Skp-Cullin-F-box (SCF) complexes and, thus, can mediate the ubiquitin-dependent degradation of signaling proteins (Hamiaux *et al.*, 2012; Zhao *et al.*, 2013).

The interaction of SLs with auxins and cytokinins in regulation of shoot lateral branching has been thoroughly studied mainly in pea (*Pisum sativum*) and Arabidopsis (for a review, see Stirnberg *et al.*, 2010; Cheng *et al.*, 2013; Rasmussen *et al.*, 2013a). Indeed, SL biosynthesis and signaling are intimately connected with auxin transport regulation (Foo *et al.*, 2005; Bennett *et al.*, 2006; Brewer *et al.*, 2009; Ferguson and Beveridge, 2009; Hayward *et al.*, 2009; Crawford *et al.*, 2010; Koltai *et al.*, 2010; Shinohara *et al.*, 2013; Pandya-Kumar *et al.*, 2014). The application of GR24 reduces the basipetal auxin transport and the accumulation of PIN-FORMED1 (PIN1) in the plasma membrane of xylem parenchyma cells in the shoot in a MAX2-dependent manner (Crawford *et al.*, 2010). Moreover, in buds, SLs promote PIN1 endocytosis through a clathrin-dependent mechanism that occurs independently of *de novo* protein synthesis (Shinohara *et al.*, 2013). In pea, SLs have been demonstrated to act also independently of auxin (Brewer *et al.*, 2015). Interestingly, SLs could inhibit shoot lateral branching only when a competing auxin source was available (Crawford *et al.*, 2010; Liang *et al.*, 2010). The auxin landscape also influences the SL control on branching, because the negative effect on shoot lateral branching disappeared and even became positive when the auxin homeostasis was changed (Shinohara *et al.*, 2013). In buds, SLs and cytokinins are known to interact antagonistically and locally (Dun *et al.*, 2012; Zhang *et al.*, 2010; Hu *et al.*, 2014), probably through their common target, BRANCHED1 (BRC1) in Arabidopsis (Minakuchi *et al.*, 2010; Braun *et al.*, 2012; Dun *et al.*, 2012).

Also in the root, the interaction of SLs with auxins has been investigated. PIN1, PIN3, and PIN7 protein levels are reduced upon prolonged treatment with GR24 (Ruyter-Spira *et al.*, 2011). Additionally, during GR24-induced root hair elongation, PIN2 abundance increases at the apical plasma membrane of epidermal cells, suggesting that SLs affect PIN2 endocytosis and endosomal trafficking via actin dynamics in a MAX2-dependent manner (Pandya-Kumar *et al.*, 2014). The inhibitory effect of GR24 on LR development can be reverted to an induction rather than a reduction of LRD by applying a high dose of auxin, or under low phosphate conditions that may increase the auxin sensitivity (Pérez-Torres *et al.*, 2008; Ruyter-Spira *et al.*, 2011). These observations suggest that, just as for branching, changes in the auxin landscape could modulate the impact of GR24 (Ruyter-Spira *et al.*, 2011).

Cytokinins are also well known to influence the root architecture (reviewed in Vanstraelen and Benková, 2012). Cytokinin signaling negatively affects LR development by impinging on PIN-dependent auxin transport (Laplaze *et al.*, 2007; Bishopp *et al.*, 2011; Marhavý *et al.*, 2011, 2014; Bielach *et al.*, 2012; Chang *et al.*, 2013; Moreira *et al.*, 2013). Interaction of SLs with cytokinins during LR development has been poorly studied, but *max2-1* mutants have been reported to have a reduced sensitivity to the synthetic cytokinin 6-benzylaminopurine (BAP) (Koren *et al.*, 2013).

Here, LR priming as well as outgrowth are shown to be modulated by treatment with GR24, the latter in a spatiotemporal manner, mainly affecting the emergence of LRs, which are closest to the root–shoot junction. In addition, the *ARABIDOPSIS HISTIDINE KINASE3* (*AHK3*)/*ARABIDOPSIS RESPONSE REGULATOR1* (*ARR1*)/*ARR12* cytokinin signaling module interacts with SLs to affect LR development, probably through changes in polar auxin transport. Altogether, the results place the SL action on LR development in the auxin landscape context via cross-talk mechanisms with cytokinin signaling.

## Materials and methods

### Plant material and growth conditions

The *pin7-1* mutant from *Arabidopsis thaliana* (L.) Heyhn. is in Landsberg *erecta* (L*er*) background, whereas the other lines described are in Columbia-0 (Col-0) background. The plant material used has been described previously: *ahk2-2*, *cre1-12*, and *ahk3-3* (Higuchi *et al.*, 2004); *ahk2;ahk3*, *ahk2;ahk4*, and *ahk3;ahk4* (Riefler *et al.*, 2006); *arr1*, *arr12*, and *arr1;arr12* (Mason *et al.*, 2005); *arr3;arr4;arr5;arr6* and *arr3;arr4;arr5;arr6;arr8;arr9* (To *et al.*, 2004); *pin1-613* (Bennett *et al.*, 2006); *35S:PIN1-GFP* (Růžička *et al.*, 2007); *pin3-3* (Friml *et al.*, 2002); *pin5-3* (Mravec *et al.*, 2009); *pin7-1* (Friml *et al.*, 2003); *shy2-24* (Tian and Reed, 1999); *proAHK3:GUS* (Higuchi *et al.*, 2004); *proPIN1:GUS* and *pGATA23:NLS-GFP-GUS* (De Rybel *et al.*, 2010); and *YUCCA1-D* (Zhao *et al.*, 2001).

Seeds were surface-sterilized for 5min in 70% (v/v) ethanol, 0.05% (v/v) SDS solution, then incubated in 95% (v/v) ethanol for 5min, and plated on half-strength Murashige and Skoog (½MS) medium [1% (w/v) sucrose and 0.8% (w/v) agar]. Plants were stratified at 4 °C for 2 d, transferred to a growth chamber at 21 °C (16-h light/8-h dark photoperiod). A racemic mixture of GR24 was supplemented to the growth medium at the start of the experiment and plants were grown for the indicated time. All the experiments were repeated three times. Chemical compounds were added in the following concentrations, except indicated otherwise: 1 μM GR24 and 0.1 μM 1-*N*-naphthylphthalamic acid (NPA).

### Phenotypic analysis and statistics

After 9 d of growth, LRs were counted under a binocular S4E microscope (Leica Microsystems) and root length was measured with ImageJ (http://rsb.info.nih.gov/ij). Both values were used to calculate the LRD. For the decapitation experiments, seedlings were grown for 6 d, whereafter the shoot was removed as described (Forsyth and Van Staden, 1981). The bottom part was transferred to ½MS medium with or without 1 µM GR24. For the complementation with indole-3-acetic acid (IAA), agar blocks (0.5cm^3^) containing solidified growth medium with and without 10 µM IAA were added to the decapitated site and the LRD was analyzed 5 d later. Replicate means were subjected to statistics by analysis of variance (ANOVA; SAS Institute Inc., Cary, NC, USA).

### Stage determination of *GATA23* expression analysis

*pGATA23:NLS-GFP-GUS* seeds were put on medium supplemented with 1 µM GR24 or with the same volume of acetone as control and were stratified for 2 d at 4 °C. Seedlings were grown vertically under continuous white light at 21 °C. At 4 d after germination (DAG), half the seedlings were harvested for analysis, whereas for the remaining seedlings, the position of the main root tip was marked and the plates were transferred back to the growth room. At 9 DAG, the root parts above the mark were harvested. Samples were stained with β-glucuronidase (GUS), cleared as described (Malamy and Benfey, 1997), and analyzed under the microscope (see below). To calculate the percentage of initiated sites, the average of initiations at 9 DAG was divided by the average sites present at 4 DAG. Likewise for the calculations of the percentage of emerged sites, the average of emerged LRs at 9 DAG was divided by that of the sites present at 4 DAG.

### Histochemical analysis of GUS activity

Whole seedlings were stained in multiwell plates as described (Jefferson *et al.*, 1987). Samples were cleared as described (Malamy and Benfey, 1997) and were analyzed by a differential interference contrast BX51 microscope (Olympus). Alternatively, samples were mounted directly in chloral hydrate solution (chloral hydrate:water:glycerol, 8:3:1) and microscopically analyzed.

### RNA isolation, quantitative RT-PCR, and statistical analysis of *PIN1* expression

Arabidopsis *pPIN1:GUS* seeds were sown on ½MS medium with or without 1 µM GR24. Seeds were stratified for 2 d at 4 °C and then grown in vertical position at 21 °C (16-h light/8-h dark photoperiod). After 7 d, root material was harvested and flash-frozen in liquid nitrogen. The region between the root–shoot junction and the first emerged LR was harvested separately from the remaining root system. Approximately 100 seedlings were used for each treatment and the experiment was repeated three times.

RNA preparation, cDNA synthesis, real-time quantitative (q)RT-PCR, and statistical analysis of expression profiling were done as described (Rasmussen *et al.*, 2013b). The primers used are the following: *PIN1*_forward GGCATGGCTATGTTCAGTCTTGGG and *PIN1*_reverse ACGGCAGGTCCAACGACAAATC; *ACTIN*_forward CGCCATCCAAGCTGTTCTC and *ACTIN*_reverse TCACGTCCAGCAAGGTCAAG.

### Accession numbers

The Arabidopsis Genome Initiative locus identifiers for the genes characterized in this study are: *AHK3* (AT1G27320), *SHY2* (AT1G04240), *PIN1* (AT1G73590), *PIN7* (AT1G23080), and *YUCCA1* (AT4G32540). Germplasm identification numbers for the seeds are: *ahk2* (*ahk2-2tk*), *ahk3-3* (SALK_069269), *cre1-12* (SALK_048970), *ahk2;ahk3* (*ahk2-5ahk3-7*), *ahk2;ahk4* (*ahk2-5cre1-12*), *ahk3;ahk4* (*ahk3-7;cre1-2*), *arr1-2* (N6368), *arr12-1* (CS6978), *arr1;arr12* (*arr1-3;arr12-1*), *pin1-613* (SALK_047613), and *pin5-3* (SALK_021738).

## Results

### GR24 reduces lateral rooting in *Arabidopsis* by affecting LR emergence, especially near the root–shoot junction in a MAX2-dependent manner

**Fig. 1.**
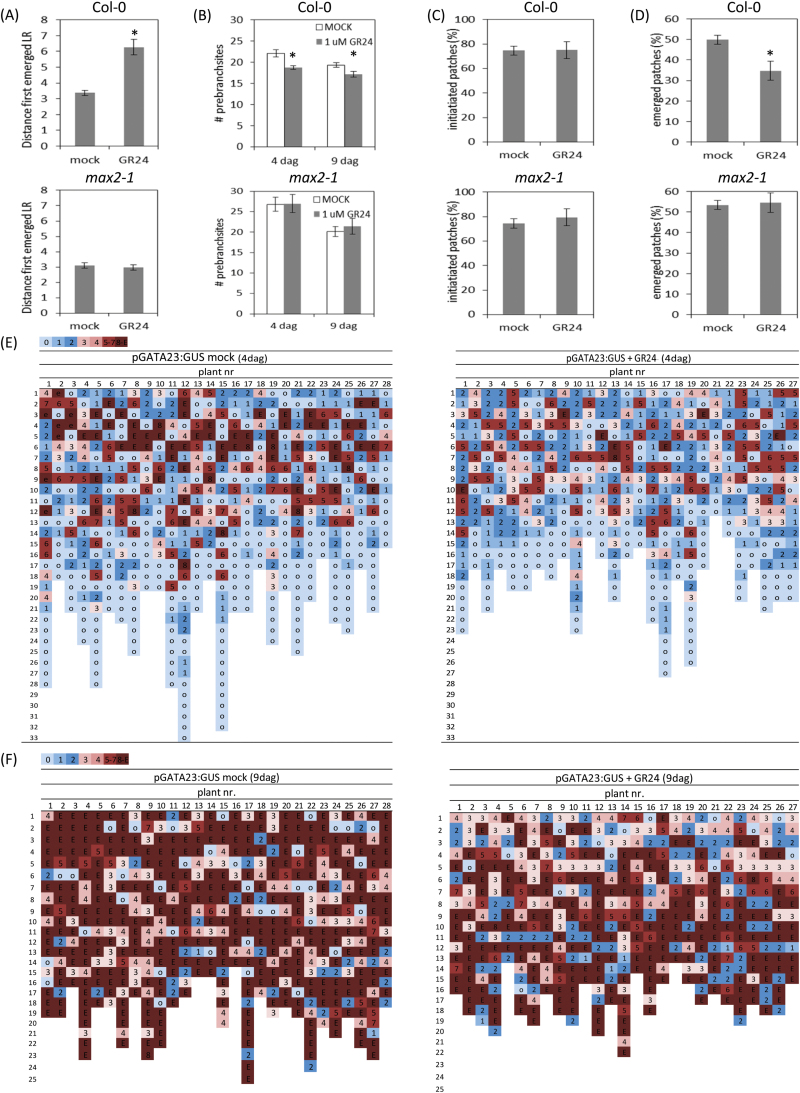
Effect of exogenous GR24 on LR development near the root–shoot junction. (A) Distance to the first emerged LR in Col-0 (top) and *max2-1* (bottom). (B) Total number of prebranch sites under mock (white bars) and GR24 treatment (gray bars), 4 and 9 DAG in Col-0 (top) and *max2-1* (bottom). (C) Percentage of initiated patches under mock and 1 μM GR24 treatments in Col-0 (top) and *max2-1* (bottom) at 9 DAG. (D) Percentage of emerged patches under mock and GR24 treatment in Col-0 (top) and *max2-1* (bottom). (A–D) Data presented are means ± standard error (SE) of three biological repeats (*n*>20). *P<0.001, according to the Student’s *t*-test. (E, F) Stages of LR primordia via *GATA23:GUS* staining in Col-0 under mock (left) and GR24 treatment (right) at 4 DAG (E) and 9 DAG (F). All events, possibly leading to emerged LRs, were scored in individual plants, color-coded, and for each plant, vertically ordered from the closest to the hypocotyl (up) downward to the meristem (down). The root fragments used for analysis were comparable in length. Data of one representative experiment are shown. The experiments were repeated three times with similar results.

The overall MAX2-dependent reduction in LRD caused by GR24 application had already been reported (Kapulnik *et al.*, 2011b; Kohlen *et al.*, 2011; Ruyter-Spira *et al.*, 2011), but phenotypical insights into this event are still lacking. Upon GR24 treatment, the first emerged LR had an altered position and this effect was abolished in the *max2-1* mutant. When plants were grown without GR24 (mock), the distance from the hypocotyl to the first emerged LR was on average 3.37mm, whereas when grown in the presence of GR24 it increased to 6.27mm in WT plants (Fig. 1A).

To understand this effect, the LR development was spatiotemporally followed, with specific focus on the upper root zone. Therefore, the expression of the early LR marker GATA23 that indicates prebranch sites (De Rybel *et al.*, 2010) was used and combined with the staging of the LR primordia (Malamy and Benfey, 1997), in both WT and *max2-1* plants, under mock and GR24 treatments (Fig. 1E, F). As such, all sites in which an LR could develop were visualized from the root–shoot junction down to the root meristem at 4 DAG (Fig. 1E; Supplementary Fig. S1A at *JXB* online). The progression in LR development was subsequently analyzed at 9 DAG (Fig. 1F; Supplementary Fig. S1B at *JXB* online) to obtain a spatiotemporal view of how the LR primordium development was affected by GR24 treatment. Fewer GATA23-marked sites were observed at 9 DAG than at 4 DAG, implying that not all primed sites developed into an LR primordium. When the number of LR sites between mock and GR24-grown plants was compared, slightly, but significantly, fewer sites were counted upon GR24 treatment, both at 4 and 9 DAG (Fig. 1B), indicating that GR24 treatment reduced the total number of prebranch sites in WT, but not in *max2-1*, seedlings (Fig. 1B). Concerning initiated patches (see Materials and Methods), mock and GR24-grown roots of both WT and *max2-1* seedlings did not differ, suggesting that GR24 had no effect on LR initiation, once the prebranch site had been formed (Fig. 1C). GR24 treatment also affected LR outgrowth (Kapulnik *et al.*, 2011b; Kohlen *et al.*, 2011; Ruyter-Spira *et al.*, 2011). When the percentage of emerged patches was calculated, significantly fewer sites were counted on GR24-grown roots than on control roots, but again not on *max2-1* roots (Fig. 1D). Interestingly, when the emergence pattern was analyzed at 9 DAG (Fig. 1F), the LR outgrowth inhibition was most pronounced at positions 1–8, corresponding to the LR primordia closest to the root–shoot junction, but did not occur in the *max2-1* mutant (see Supplementary Fig. S1B at *JXB* online). These data indicate that mainly the first formed LR primordia, thus those near the root–shoot junction, do not develop when plants are grown in the presence of GR24 and that this effect depends on MAX2.

### The cytokinin signaling components *AHK3, ARR1*, and *ARR12* mediate the effect of GR24 on LR development

**Fig. 2.**
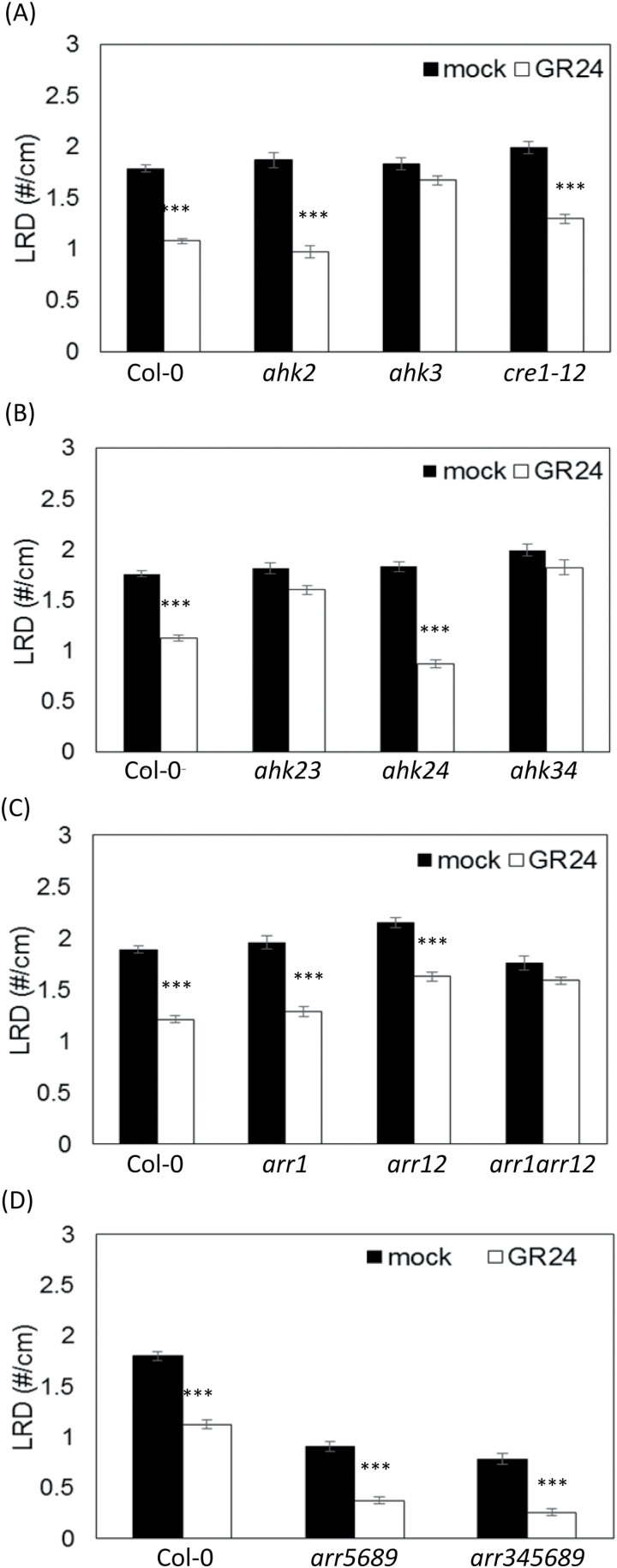
Effects of GR24 on cytokinin perception and signaling mutants. LRD of single cytokinin receptor mutants (A), double cytokinin receptor mutants (B), B-type response regulators *ARR1*, *ARR12*, and *ARR1;ARR12* (C), and mutants in higher-order A-type response regulators (D) upon GR24 treatment. Data presented are means ± SE of three biological repeats (*n*>20). ***P<0.001, according to ANOVA mixed-model statistical analyses.

Both cytokinins and SLs have been described as negative regulators of LR development in Arabidopsis (Benková *et al.*, 2003; Li *et al.*, 2006; Laplaze *et al.*, 2007; Kapulnik *et al.*, 2011b; Ruyter-Spira *et al.*, 2011). Therefore, the link between the GR24-mediated LRD reduction and the cytokinin-mediated LRD inhibition was investigated in further detail. First, the LRD of several cytokinin signaling mutants, single and higher-order mutants affected in the cytokinin receptors *CYTOKININ RESPONSE1 (CRE1)/AHK4*, *AHK2*, and/or *AHK3* (see Materials and Methods) was examined upon treatment with 1 µM GR24 (Fig. 2A, B). For all tested genotypes, GR24 treatment did not significantly affect the main root length (see Supplementary Fig. S2 at *JXB* online). For Col-0, *cre1/ahk4*, and *ahk2*, the LRD was significantly reduced upon GR24 treatment, but not for the *ahk3* mutant (Fig. 2A). In the double cytokinin receptor mutant *ahk2;ahk4*, the LRD decreased significantly upon GR24 treatment, whereas no significant changes in LRD were observed for *ahk2;ahk3* and *ahk3;ahk4* between mock and GR24 treatment (Fig. 2B). Taken together, these data show that in mutants specifically affected in one member of the cytokinin receptor family, i.e. *AHK3* (*ahk3*, *ahk2;ahk3*, and *ahk3;ahk4*), the GR24 impact on LRD was abolished, whereas other cytokinin receptor mutants responded as WT plants. The *AHK3* expression was unaffected by GR24 treatment (see Supplementary Fig. S3 at *JXB* online).

These observations prompted the investigation of the downstream signaling components of the cytokinin perception machinery. As the B-type response regulators *ARR1* and *ARR12* are involved in mediating the *AHK3*-dependent effects in the root elongation zone (Dello Ioio *et al.*, 2007, 2008), the GR24 impact on the LRD was tested in mutants of these response regulators. The single mutants *arr1* and *arr12* displayed a sensitivity to GR24 similar to that of Col-0 (Fig. 2C), but the double mutant *arr1;arr12* did not, indicating that both ARRs need to be disrupted to interfere with the GR24 effect on LR development (Fig. 2C).

Having established that *AHK3*, *ARR1*, and *ARR12* are involved in the GR24-mediated reduction of LRD, we analyzed whether mutants affected in A-type response regulators would affect the GR24-mediated LRD reduction. Therefore, the sensitivity was tested of higher-order A-type ARR mutants to GR24, because these negative regulators of the cytokinin response are known to act redundantly in root architecture control (To *et al.*, 2004; Zhang *et al.*, 2011). The *arr5;arr6;arr8;arr9* and *arr3;arr4;arr5;arr6;arr8;arr9* mutants showed a significant increase in sensitivity to GR24: LRD decreased by 37% in WT and by 58% and 67% in *arr5;arr6;arr8;arr9* and *arr3;arr4;arr5;arr6;arr8;arr9*, respectively (Fig. 2D). Hence, these data support the hypothesis that an altered cytokinin responsiveness can enhance (A-type ARRs) or repress (B-type ARRs or AHK3) the GR24 effect on LR development. Taken together, these experiments demonstrate that specific cytokinin signaling components are needed for the GR24 action on LR development.

### The modified sensitivity to GR24 of *ahk3/arr1;arr12/shy2* mutants is due to changes in the auxin landscape

**Fig. 3.**
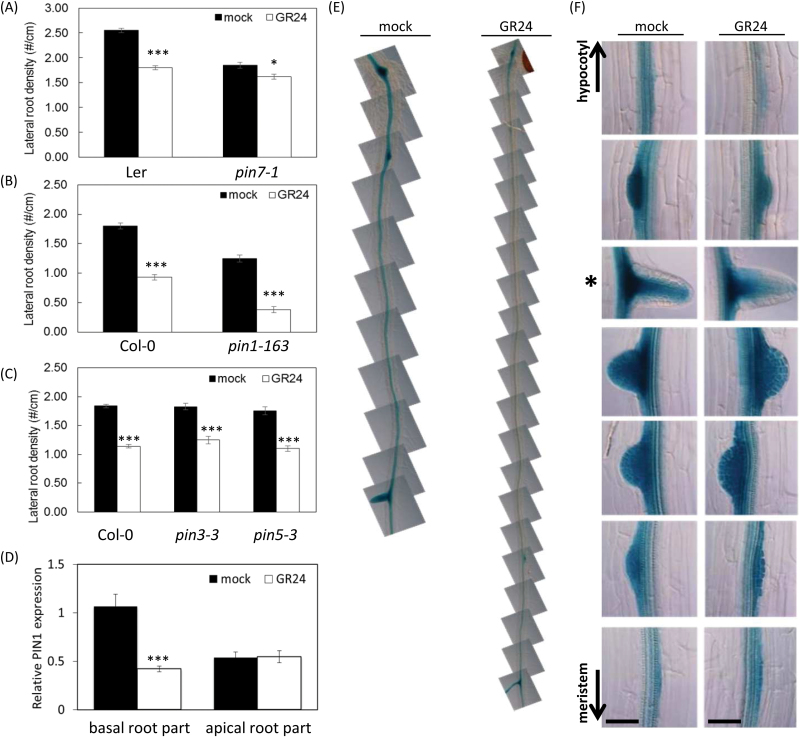
Interrelation between the polar auxin transport and the GR24 effect on LR development. (A–C) LRD of *pin7-1*, *pin1-613*, *pin3-3*, and *pin5-3* mutants compared with WT grown in the presence or absence of GR24. Data presented are means ± SE of three biological repeats (*n*>20). (D) Relative *PIN1* expression in 5-d-old seedlings under mock and GR24 treatment as determined by qRT-PCR. Material was harvested separately from the upper part (old, above the first emerged LR) and the lower part (young) of the root. ***P<0.001, *P<0.05, according to ANOVA mixed-model statistical analyses.(E) p*PIN1:GUS* expression patterns of plants grown with and without *GR24*, 7 d after growth. Frames until the first emerged LR are shown. (F) Expression of *PIN1* with p*PIN1:GUS* plants during different stages of LR development under mock and GR24 treatment. The panels indicated by the asterisk display the first emerged LR and those above the asterisk correspond to the LR primordia near the root–shoot junction. Scale bars=40 µm.

The AHK3/ARR1/ARR12 cytokinin signaling pathway has been shown to act upstream of *SHORT HYPOCOTYL2* (*SHY2*) to control root differentiation (Dello Ioio *et al.*, 2007, 2008) and, additionally, the *shy2* loss-of-function mutant to be insensitive to GR24 as well (Koren *et al.*, 2013). To elucidate why mutants in the *AHK3/ARR1/ARR12/SHY2* module are affected in their GR24 sensitivity, the GR24 phenotype of different *pin* mutants was examined, because *SHY2* specifically represses *PIN1*, *PIN3*, *PIN5*, and *PIN7* and cytokinin treatment downregulates *PIN1* and *PIN3*, but upregulates *PIN7* expression (Dello Ioio *et al.*, 2007; Růžička *et al.*, 2009). First, the GR24 effect on LRD of mutations in *PIN1*, *PIN3*, *PIN5*, or *PIN7* was analyzed. The decrease in LRD of the *pin7* mutants was only minor upon GR24 treatment, indicating that mutations in *PIN7* reduced the root sensitivity to GR24 (Fig. 3A); however, the LRD reduction of the *pin1-613* mutants was significantly higher than that in WT plants (Fig. 3B). For the *pin3-3* and *pin5-3* mutants, the LRD did not differ from that of WT plants (Fig. 3C).

Hence, these results provide the first genetic evidence that the LR response to exogenous GR24 is modulated by interference with the polar auxin transport through PIN1 and, to a lesser extent, PIN7. Previously, prolonged, but not short, GR24 treatments had been demonstrated to influence the expression of *PIN1*, *PIN3*, and *PIN7* in the root meristem, but the expression in root parts other than the meristem had not been assessed (Ruyter-Spira *et al.*, 2011; Shinohara *et al.*, 2013). Therefore, the GR24 effect was investigated on the transcription of *PIN1* in the mature root, at the hypocotyl–root junction, where LR emergence is most affected by the GR24 treatment (Fig. 3). The GR24 impact on *PIN1* expression was analyzed after 7 d of growth of p*PIN1:GUS* seedlings. Interestingly, *PIN1* expression was affected in a spatial way because, especially closest to the shoot, the expression in the vasculature was lower upon GR24 treatment than under mock conditions (Fig. 3E, F). This observation was confirmed by analyzing the *PIN1* gene expression by qRT-PCR of roots grown either in the presence or the absence of GR24 and by assessing the mature versus younger regions of the root (Fig. 3D). Moreover, *PIN1* expression was also lower in the developing LRs from the upper part of GR24-treated plants than that of mock-grown roots, in contrast to developing LRs at younger stages, i.e. near the root meristem (Fig. 3F).

Thus far, our data demonstrate that mutations in the *AHK3*/*ARR1/ARR12* cytokinin signaling module and in the auxin transport genes *PIN1* and *PIN7* affect the root sensitivity to GR24, and that GR24 influences auxin homeostasis by downregulating the expression of *PIN1* near the shoot–root junction, in agreement with the decreased PIN protein levels in the root upon prolonged treatments with high concentrations of GR24 (Ruyter-Spira *et al.*, 2011).

**Fig. 4.**
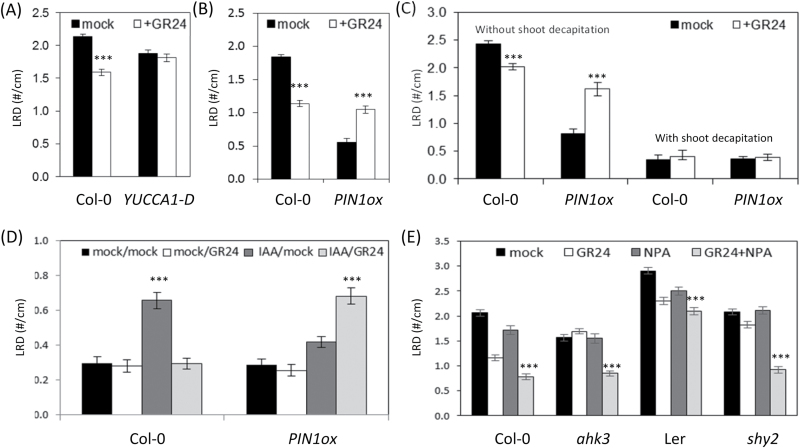
Dependence of GR24 action on the plant auxin status. (A) LRD of WT and *YUCCA*-overexpressing (*YUCCA1-D*) plants, grown with and without GR24. (B) LRD of WT and *PIN1*-overexpressing (*PIN1ox*) plants, grown with and without GR24. (C) LRD of Col-0 and *35S:PIN1* (*PIN1ox*) plants with and without shoot decapitation, grown in the presence or absence of GR24. (D) LRD of Col-0 and *PIN1ox* plants with decapitation and with and without apically applied IAA grown in the presence or absence of GR24. Mock/mock: decapitated plants grown in the absence of GR24 and without applied IAA; mock/+GR24: decapitated plants grown in the presence of GR24 and without applied IAA; IAA/mock: decapitated plants grown in the absence of GR24 and with apically applied IAA; IAA/+GR24: decapitated plants grown in the presence of GR24 and with apically applied IAA. (E) LRD of Col-0, *ahk3,* L*er* and *shy2-24* mutants upon treatment with mock, GR24, NPA, or NPA+GR24. Data presented are means ± SE of three biological repeats (*n*>20). ***P<0.001, according to ANOVA mixed-model statistical analyses.

To further investigate how the auxin environment alters the GR24 effect, the GR24 response was examined in plants that overexpressed *YUCCA* with concomitantly increased free auxin levels (Zhao *et al.*, 2001). The LRD of *YUCCA1-D* plants did not decrease upon GR24 treatment, indicating that enhanced endogenous auxin levels also modulate the GR24 response in roots (Fig. 4A). Also *35S:PIN1*-overexpressing plants that have highly increased frequencies of root primordia with retarded growth were analyzed (Benková *et al.*, 2003). The typical GR24-mediated decrease in LRD was no longer visible, but rather an increase in LRD (Fig. 4B). Moreover, when the foliar auxin source that determines the outgrowth potential of LRs (Bhalerao *et al.*, 2002; Ljung *et al.*, 2005) was removed by decapitation after 6 d of growth and when these plants were subsequently treated with GR24 for 5 d, the effects disappeared on both the *PIN1*-overpressing lines (increase in LRD) and the WT (decrease in LRD), indicating that shoot-derived auxin is important for the GR24 responses in roots (Fig. 4D). Application of IAA in these experiments (see Materials and Methods) revealed that shoot-derived auxin mediated the effect, because it complemented the phenotype of decapitated plants (Fig. 4D). Altogether, the functional data demonstrate that shoot-derived auxin controls the effect of GR24 on lateral rooting in Arabidopsis, as previously hypothesized (Ruyter-Spira *et al.*, 2011).

All mutants with GR24-insensitive root responses, i.e. *ahk3*, *arr1;arr12*, and *shy2-24*, display enhanced *PIN1* expression (Dello Ioio *et al.*, 2007, 2008; Zhang *et al.*, 2011) that might cause their insensitivity toward GR24. This hypothesis was tested by applying low concentrations (100nM) of NPA, a polar auxin transport inhibitor (Himanen *et al.*, 2002). The LRD response was analyzed under mock and GR24 treatment after 9 d of growth (Fig. 4E). Addition of this low concentration of NPA had no impact on the LRD (Fig. 4E) and had no clear effect on *PIN1* expression in the main root, although a slight increase in *PIN1* gene expression was observed in the root tip (see Supplementary Fig. S4 at *JXB* online). However, when the *ahk3* and *shy2-24* mutants were grown on plates supplemented with NPA as well as GR24, the LRD was lower than that of roots grown under mock conditions or supplemented with GR24 or NPA alone, implying that treatment with NPA rendered the mutant plants responsive to GR24 again. For Col-0, no additional effect was seen when the roots were treated with both NPA and GR24.

## Discussion

**Fig. 5.**
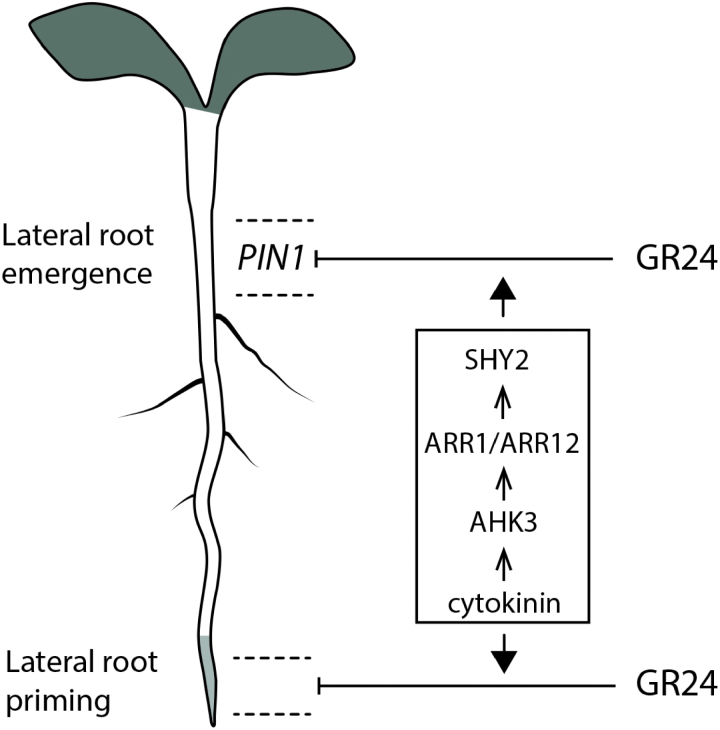
Working model on the interaction of cytokinins with the SL analog GR24 to control LR development. GR24 treatment results in inhibition of LR emergence, mainly, but not exclusively, near the root–shoot junction and, to a minor extent, in inhibition of LR priming in the root meristem zone. In the root region near the root–shoot junction, this LR emergence inhibition coincides with a spatial downregulation of *PIN1* expression by GR24 treatment. The cytokinin module that signals via AHK3, through the response regulators ARR1/ARR12, and ultimately to SHY2, influences the effect of GR24 on LR development. Mutants in this pathway are insensitive to GR24, probably due to their reported increased PIN1 levels, because reduction of the auxin flux by NPA treatment renders the mutants sensitive again to GR24.

Several aspects of the root system architecture are modulated by SLs (for reviews, see Cheng *et al.*, 2013; Rasmussen *et al.*, 2013a; Koltai, 2014). Here, GR24 was found to control LR development spatiotemporally and to interplay with cytokinin that, just like SLs, regulates LR development. A summarizing model is presented (Fig. 5).

The method established to build a developmental map of all possible initiated LRs combines the *GATA23* marker gene for induction of prebranching sites, i.e. pericycle-derived LR founder cells that are predestined to start cell division for LR development and LR positioning (Malamy and Benfey, 1997; De Rybel *et al.*, 2010). Together with the determination of the position of each event along the main root, a precise developmental map provides location and developmental stage of each LR event, thereby revealing that the main effect of GR24 on the development of LRs concerns their emergence. This observation concurs with previously published work, although the proposed specific interruption at stage V of LR development was not detected (Ruyter-Spira *et al.*, 2011).

On the 9-DAG map, the LRs were mainly, but not exclusively, situated close to the root–shoot junction that no longer emerged under GR24 treatment. Accordingly, the distance between the hypocotyl–shoot junction and the first emerged LR was longer in GR24-grown roots than in control roots. This MAX2-dependent effect concurs with its essential function in SL signaling. Hence, GR24 might affect specifically the emergence of the LRs that develop first and are positioned in the older part of the root. This spatiotemporal effect was also seen on the *PIN1* expression pattern in the root. Although the reason for this effect still needs to be investigated, the disappearance of the SL receptor might be the underlying cause, because GR24 treatment reduces D14 protein abundance in roots (Chevalier *et al.*, 2014).

Additionally, a small, but significant, decrease in prebranch sites was visible, whereas GR24 had no appreciable impact on LR initiation. The previously detected GR24 effect on LR initiation (Kapulnik *et al.*, 2011b; Ruyter-Spira *et al.*, 2011) might be due to an impact on prebranching. Prebranch sites are established by a periodic oscillation of auxin concentrations accompanied by fluctuations in specific gene expression (De Smet *et al.*, 2007; Moreno-Risueno *et al.*, 2010). This oscillating pattern has been found to be mediated by a carotenoid compound, distinct from SLs (Van Norman *et al.*, 2014). In agreement with the data presented, the *max2* mutants also exhibited an increased LR capacity (Van Norman *et al.*, 2014). It would be interesting to analyze whether GR24, as a mimic of SLs or related compounds, modulates the periodic oscillation of auxin to cause the small effect on prebranching. Furthermore, independently of SLs, at 9 DAG, fewer LR events are observed on the same main root part than at 4 DAG, possibly indicating that not all primed sites develop into LRs.

Cytokinins have been identified as endogenous repressors of LR development in a close interplay with auxin (Benková *et al.*, 2003; Li *et al.*, 2006; Laplaze *et al.*, 2007). Here, the GR24 effect on LR development required the functional cytokinin receptor AHK3, but not AHK2 and AHK4/CRE1. The dependence on *AHK3* and not on *AHK4* is remarkable, because *AHK4* has been implicated in LR patterning along the main root (Marhavý *et al.*, 2011), whereas *AHK3* and the two immediately downstream B-type response regulator genes, *ARR1* and *ARR12*, play an important role in determining the root meristem size (Dello Ioio *et al.*, 2007, 2008). Also in the experimental setup, the double mutant *arr1;arr12* had no LR response toward GR24, implying that the same cytokinin module (*AHK3/ARR1/ARR12*) that determines the root meristem differentiation also governs the GR24 action on LR development. *AHK3* is involved in meristem differentiation by transcriptional control of the auxin-induced *SHY2/IAA3* gene (Dello Ioio *et al.*, 2007, 2008). The typical reduction in lateral rooting upon GR24 treatment was indeed not seen in the *shy2-24* loss-of-function mutants (Koren *et al.*, 2013), supporting the hypothesis that the *AHK3/ARR1/ARR12* module acts through *SHY2* to result in GR24 insensitivity.

The *AHK3/ARR1/ARR12/SHY2* module negatively influences *PIN1/PIN3/PIN5/PIN7* expression (Dello Ioio *et al.*, 2007, 2008), whereas cytokinin treatment downregulates *PIN1/PIN3/PIN5*, but upregulates *PIN7* expression (Laplaze *et al.*, 2007; Růžička *et al.*, 2009). These changes in *PIN* gene expression and their consequences on polar auxin transport might be the underlying cause for the GR24 insensitivity of the mutants. Several *PIN* mutants had a modified sensitivity to GR24: *pin3* and *pin5* mutants still displayed a reduced LR development upon GR24 treatment, whereas *pin7* mutants were only slightly responsive to GR24, and *pin1-613* mutants were hypersensitive, in agreement with the opposite influence of cytokinins on their expression. In addition, treatment of *ahk3* and *shy2-24* with NPA made them sensitive again to GR24. Hence, the changes in *PIN* gene expression, such as the *PIN1* overexpression observed in these mutants (Dello Ioio *et al.*, 2007, 2008; Zhang *et al.*, 2011) with an enhanced polar auxin transport as a result, might be the reason that GR24 does not reduce the LRD in these mutants.

Moreover, the data support the central role of auxin transport for SL action. Based on exogenous auxin and phosphate level modulation, the auxin content in roots has been shown to determine its responsiveness toward GR24 (Ruyter-Spira *et al.*, 2011). Indeed, endogenous overproduction of auxin via overexpression of *YUCCA* could make LR development unresponsive to GR24. As auxin is well known to positively regulate its own efflux from cells, PIN1 internalization in the *YUCCA1-D* mutant was reduced, resulting in the accumulation of PIN1 on the plasma membrane (Paciorek *et al.*, 2005), an observation fitting the theory that mutants with an enhanced *PIN1* expression are insensitive to GR24. Interestingly, *PIN1*-overexpressing plants no longer displayed a reduced LRD when treated with GR24, but an opposite phenotype with an increased LRD. The difference in phenotypes between the plants overexpressing *YUCCA1-D* and *PIN1* is intriguing, but might be due to differences in the severity of PIN1 accumulation. Also in the shoot, depending on the auxin transport landscape, GR24 could have positive or negative effects on the shoot lateral branching by depleting PIN1 from the membranes of xylem parenchyma cells of inflorescence stems (Shinohara *et al.*, 2013). In addition, GR24 has been shown to have a different impact on LR development that depends on the growth conditions: inhibition under sufficient and induction under low phosphate conditions or with exogenous IAA (Ruyter-Spira *et al.*, 2011). Hence, *PIN1* overexpression has an effect on GR24 responses similar to that of phosphate-limiting conditions: an increase, rather than a decrease, in LRD.

In conclusion, the data presented imply that GR24 regulates LR development in a spatiotemporal manner with the strongest effect on emergence of the first developed LR positioned close to the root–shoot junction. This effect is tightly integrated into the auxin–cytokinin network that rules the root architecture, with the polar auxin transport capacity as a central player on which both cytokinin and GR24 act.

## Supplementary data

Supplementary data are available at *JXB* online.

Fig. S1. Stages of LR primordia via p*GATA23:GUS* staining in *max2-1* under mock and GR24 treatment at 4 and 9 DAG.

Fig. S2. Main root lengths of WT and cytokinin receptor and signal transduction mutants under mock and GR24 treatment.

Fig. S3. p*AHK3:GUS* expression patterns of LR primordia at different developmental stages under mock and GR24 treatment.

Fig. S4. p*PIN1:GUS* expression pattern after treatment with 0.1 µM NPA around the root–shoot junction (left) and the root meristem zone (right).

Supplementary Data

## Abbreviations:

AHK: ARABIDOPSIS HISTIDINE KINASE
ARR: ARABIDOPSIS RESPONSE REGULATOR
BAP: 6-benzylaminopurine
BES: BRASSINOSTEROID INSENSITIVE--EMS-SUPPRESSOR
BRC: BRANCHED
CRE: CYTOKININ RESPONSE
D14: DWARF14
DAG: days after germination
EMS: ethyl methanesulfonate
GUS: β-glucuronidase
IAA: indole-3-acetic acid
LR: lateral root
LRD: lateral root density
MAX: MORE AXILLARY GROWTH
NPA: 1-*N*-naphthylphthalamic acid
PIN: PIN-FORMED
SCF: Skp-Cullin-F-box
SHY: SHORT HYPOCOTYL
SL: strigolactone
WT: wild-type
XPP: xylem pole pericycle.

## References

[CIT0001] Agusti J Herold S Schwarz M 2011 Strigolactone signaling is required for auxin-dependent stimulation of secondary growth in plants. Proceedings of the National Academy of Sciences, USA 108, 20242–20247 [*Erratum Proceedings of the National Academy of Sciences, USA* **109**, 14277]. 10.1073/pnas.1111902108 PMC3250165 22123958

[CIT0002] Benková E Michniewicz M Sauer M Teichmann T Seifertová D Jürgens G Friml J 2003 Local, efflux-dependent auxin gradients as a common module for plant organ formation. Cell 115, 591–602. 14651850 10.1016/s0092-8674(03)00924-3

[CIT0003] Bennett T Sieberer T Willett B Booker J Luschnig C Leyser O 2006 The *Arabidopsis MAX* pathway controls shoot branching by regulating auxin transport. Current Biology 16, 553–563. 16546078 10.1016/j.cub.2006.01.058

[CIT0004] Bhalerao RP Eklöf J Ljung K Marchant A Bennett M Sandberg G 2002 Shoot-derived auxin is essential for early lateral root emergence in *Arabidopsis* seedlings. Plant Journal 29, 325–332. 11844109 10.1046/j.0960-7412.2001.01217.x

[CIT0005] Bielach A Duclercq J Marhavý P Benková E 2012 Genetic approach towards the identification of auxin-cytokinin crosstalk components involved in root development. Philosophical Transactions of the Royal Society B-Biological Sciences 367, 1469–1478. 10.1098/rstb.2011.0233 PMC3321684 22527389

[CIT0006] Bishopp A Benková E Helariutta Y 2011 Sending mixed messages: auxin-cytokinin crosstalk in roots. Current Opinion in Plant Biology 14, 10–16. 20926335 10.1016/j.pbi.2010.08.014

[CIT0007] Braun N de Saint Germain A Pillot J-P 2012 The pea TCP transcription factor PsBRC1 acts downstream of strigolactones to control shoot branching. Plant Physiology 158, 225–238. 22045922 10.1104/pp.111.182725 PMC3252107

[CIT0008] Brewer PB Dun EA Ferguson BJ Rameau C Beveridge CA 2009 Strigolactone acts downstream of auxin to regulate bud outgrowth in pea and Arabidopsis. Plant Physiology 150, 482–493. 19321710 10.1104/pp.108.134783 PMC2675716

[CIT0009] Brewer PB Dun EA Gui R Mason MG Beveridge CA 2015 Strigolactone inhibition of branching independent of polar auxin transport. Plant Physiology 168, 1820–1829. 26111543 10.1104/pp.15.00014 PMC4528729

[CIT0010] Bu Q Lv T Shen H 2014 Regulation of drought tolerance by the F-box protein MAX2 in Arabidopsis. Plant Physiology 164, 424–439. 24198318 10.1104/pp.113.226837 PMC3875819

[CIT0011] Chang L Ramireddy E Schmülling T 2013 Lateral root formation and growth of *Arabidopsis* is redundantly regulated by cytokinin metabolism and signalling genes. Journal of Experimental Botany 64, 5021–5032. 24023250 10.1093/jxb/ert291 PMC3830484

[CIT0012] Cheng X Ruyter-Spira C Bouwmeester H 2013 The interaction between strigolactones and other plant hormones in the regulation of plant development. Frontiers in Plant Science 4, 199. 23785379 10.3389/fpls.2013.00199 PMC3683633

[CIT0013] Chevalier F Nieminen K Sánchez-Ferrero JC Rodríguez ML Chagoyen M Hardtke CS Cubas P 2014 Strigolactone promotes degradation of DWARF14, an α/β hydrolase essential for strigolactone signaling in *Arabidopsis* . Plant Cell 26, 1134–1150. 24610723 10.1105/tpc.114.122903 PMC4001374

[CIT0014] Crawford S Shinohara N Sieberer T Williamson L George G Hepworth J Müller D Domagalska MA Leyser O 2010 Strigolactones enhance competition between shoot branches by dampening auxin transport. Development 137, 2905–2913. 20667910 10.1242/dev.051987

[CIT0015] De Rybel B Vassileva V Parizot B 2010 A novel Aux/IAA28 signaling cascade activates GATA23-dependent specification of lateral root founder cell identity. Current Biology 20, 1697–1706. 20888232 10.1016/j.cub.2010.09.007

[CIT0016] De Smet I Tetsumura T De Rybel B 2007 Auxin-dependent regulation of lateral root positioning in the basal meristem of *Arabidopsis* . Development 134, 681–690. 17215297 10.1242/dev.02753

[CIT0017] Dello Ioio R Scaglia Linhares F Scacchi E Casamitjana-Martinez E Heidstra R Costantino P Sabatini S 2007 Cytokinins determine *Arabidopsis* root-meristem size by controlling cell differentiation. Current Biology 17, 678–682. 17363254 10.1016/j.cub.2007.02.047

[CIT0018] Dello Ioio R Nakamura K Moubayidin L Perilli S Taniguchi M Morita MT Aoyama T Costantino P Sabatini S 2008 A genetic framework for the control of cell division and differentiation in the root meristem. Science 322, 1380–1384. 19039136 10.1126/science.1164147

[CIT0019] Dun EA de Saint Germain A Rameau C Beveridge CA 2012 Antagonistic action of strigolactone and cytokinin in bud outgrowth control. Plant Physiology 158, 487–498. 22042819 10.1104/pp.111.186783 PMC3252097

[CIT0020] Ferguson BJ Beveridge CA 2009 Roles for auxin, cytokinin, and strigolactone in regulating shoot branching. Plant Physiology 149, 1929–1944. 19218361 10.1104/pp.109.135475 PMC2663762

[CIT0021] Foo E Bullier E Goussot M Foucher F Rameau C Beveridge CA 2005 The branching gene *RAMOSUS1* mediates interactions among two novel signals and auxin in pea. Plant Cell 17, 464–474. 15659639 10.1105/tpc.104.026716 PMC548819

[CIT0022] Forsyth C Van Staden J 1981 The effects of root decapitation on lateral root formation and cytokinin production in Pisum sativum. Physiologia Plantarum 51, 375–379.

[CIT0023] Friml J Wiśniewska J Benková E Mendgen K Palme K 2002 Lateral relocation of auxin efflux regulator PIN3 mediates tropism in *Arabidopsis* . Nature 415, 806–809. 11845211 10.1038/415806a

[CIT0024] Friml J Vieten A Sauer M Weijers D Schwarz H Hamann T Offringa R Jürgens G 2003 Efflux-dependent auxin gradients establish the apical–basal axis of *Arabidopsis* . Nature 426, 147–153. 14614497 10.1038/nature02085

[CIT0025] Gomez-Roldan V Fermas S Brewer PB 2008 Strigolactone inhibition of shoot branching. Nature 455, 189–194. 18690209 10.1038/nature07271

[CIT0026] Hamiaux C Drummond RSM Janssen BJ Ledger SE Cooney JM Newcomb RD Snowden KC 2012 DAD2 is an α/β hydrolase likely to be involved in the perception of the plant branching hormone, strigolactone. Current Biology 22, 2032–2036. 22959345 10.1016/j.cub.2012.08.007

[CIT0027] Hayward A Stirnberg P Beveridge C Leyser O 2009 Interactions between auxin and strigolactone in shoot branching control. Plant Physiology 151, 400–412. 19641034 10.1104/pp.109.137646 PMC2735998

[CIT0028] Higuchi M Pischke MS Mähönen AP 2004 *In planta* functions of the *Arabidopsis* cytokinin receptor family. Proceedings of the National Academy of Sciences, USA 101, 8821–8826. 10.1073/pnas.0402887101 PMC423279 15166290

[CIT0029] Himanen K Boucheron E Vanneste S de Almeida Engler J Inzé D Beeckman T 2002 Auxin-mediated cell cycle activation during early lateral root initiation. Plant Cell 14, 2339–2351. 12368490 10.1105/tpc.004960 PMC151221

[CIT0030] Hu Z Yamauchi T Yang J 2014 Strigolactone and cytokinin act antagonistically in regulating rice mesocotyl elongation in darkness. Plant and Cell Physiology 55, 30–41. 24151204 10.1093/pcp/pct150

[CIT0031] Jefferson RA Kavanagh TA Bevan MW 1987 GUS fusions: β-glucuronidase as a sensitive and versatile gene fusion marker in higher plants. EMBO Journal 6, 3901–3907. 3327686 10.1002/j.1460-2075.1987.tb02730.x PMC553867

[CIT0032] Kapulnik Y Resnick N Mayzlish-Gati E Kaplan Y Wininger S Hershenhorn J Koltai H 2011a Strigolactones interact with ethylene and auxin in regulating root-hair elongation in *Arabidopsis* . Journal of Experimental Botany 62, 2915–2924. 21307387 10.1093/jxb/erq464

[CIT0033] Kapulnik Y Delaux P-M Resnick N 2011b Strigolactones affect lateral root formation and root-hair elongation in *Arabidopsis* . Planta 233, 209–216. 21080198 10.1007/s00425-010-1310-y

[CIT0034] Kohlen W Charnikhova T Liu Q Bours R Domagalska MA Beguerie S Verstappen F Leyser O Bouwmeester H Ruyter-Spira C 2011 Strigolactones are transported through the xylem and play a key role in shoot architectural response to phosphate deficiency in nonarbuscular mycorrhizal host Arabidopsis. Plant Physiology 155, 974–987. 21119045 10.1104/pp.110.164640 PMC3032481

[CIT0035] Koltai H 2014 Receptors, repressors, PINs: a playground for strigolactone signaling. Trends in Plant Science 19, 727–733. 25037847 10.1016/j.tplants.2014.06.008

[CIT0036] Koltai H Dor E Hershenhorn J 2010 Strigolactones’ effect on root growth and root-hair elongation may be mediated by auxin-efflux carriers. Journal of Plant Growth Regulation 29, 129–136.

[CIT0037] Koren D Resnick N Mayzlish Gati E Belausov E Weininger S Kapulnik Y Koltai H 2013 Strigolactone signaling in the endodermis is sufficient to restore root responses and involves SHORT HYPOCOTYL 2 (SHY2) activity. New Phytologist 198, 866–874. 23425316 10.1111/nph.12189

[CIT0038] Laplaze L Benkova E Casimiro I 2007 Cytokinins act directly on lateral root founder cells to inhibit root initiation. Plant Cell 19, 3889–3900. 18065686 10.1105/tpc.107.055863 PMC2217640

[CIT0039] Li X Mo X Shou H Wu P 2006 Cytokinin-mediated cell cycling arrest of pericycle founder cells in lateral root initiation of *Arabidopsis* . Plant and Cell Physiology 47, 1112–1123. 16854941 10.1093/pcp/pcj082

[CIT0040] Liang J Zhao L Challis R Leyser O 2010 Strigolactone regulation of shoot branching in chrysanthemum (*Dendranthema grandiflorum*). Journal of Experimental Botany 61, 3069–3078. 20478970 10.1093/jxb/erq133 PMC2892150

[CIT0041] Ljung K Hull AK Celenza J Yamada M Estelle M Normanly J Sandberg G 2005 Sites and regulation of auxin biosynthesis in Arabidopsis roots. Plant Cell 17, 1090–1104. 15772288 10.1105/tpc.104.029272 PMC1087988

[CIT0042] Malamy JE Benfey PN 1997 Organization and cell differentiation in lateral roots of *Arabidopsis thaliana* . Development 124, 33–44. 9006065 10.1242/dev.124.1.33

[CIT0043] Marhavý P Bielach A Abas L 2011 Cytokinin modulates endocytic trafficking of PIN1 auxin efflux carrier to control plant organogenesis. Developmental Cell 21, 796–804. 21962902 10.1016/j.devcel.2011.08.014

[CIT0044] Marhavý P Duclercq J Weller B Feraru E Bielach A Offringa R Friml J Schwechheimer C Murphy A Benková E 2014 Cytokinin controls polarity of PIN1-dependent auxin transport during lateral root organogenesis. Current Biology 24, 1031–1037. 24768050 10.1016/j.cub.2014.04.002

[CIT0045] Mason MG Mathews DE Argyros DA Maxwell BB Kieber JJ Alonso JM Ecker JR Schaller GE 2005 Multiple type-B response regulators mediate cytokinin signal transduction in *Arabidopsis* . Plant Cell 17, 3007–3018. 16227453 10.1105/tpc.105.035451 PMC1276026

[CIT0046] Mayzlish-Gati E De-Cuyper C Goormachtig S 2012 Strigolactones are involved in root response to low phosphate conditions in Arabidopsis. Plant Physiology 160, 1329–1341. 22968830 10.1104/pp.112.202358 PMC3490576

[CIT0047] Minakuchi K Kameoka H Yasuno N 2010 *FINE CULM1* (*FC1*) works downstream of strigolactones to inhibit the outgrowth of axillary buds in rice. Plant and Cell Physiology 51, 1127–1135. 20547591 10.1093/pcp/pcq083 PMC2900823

[CIT0048] Moreira S Bishopp A Carvalho H Campilho A 2013 AHP6 inhibits cytokinin signaling to regulate the orientation of pericycle cell division during lateral root initiation. PLoS ONE , 8, e56370. 23457561 10.1371/journal.pone.0056370 PMC3572949

[CIT0049] Moreno-Risueno MA Van Norman JM Moreno A Zhang J Ahnert SE Benfey PN 2010 Oscillating gene expression determines competence for periodic *Arabidopsis* root branching. Science 329, 1306–1311. 20829477 10.1126/science.1191937 PMC2976612

[CIT0050] Mravec J Skůpa P Bailly A 2009 Subcellular homeostasis of phytohormone auxin is mediated by the ER-localized PIN5 transporter. Nature 459, 1136–1140. 19506555 10.1038/nature08066

[CIT0051] Paciorek T Zažímalová E Ruthardt N 2005 Auxin inhibits endocytosis and promotes its own efflux from cells. Nature 435, 1251–1256. 15988527 10.1038/nature03633

[CIT0052] Pandya-Kumar N Shema R Kumar M 2014 Strigolactone analog GR24 triggers changes in PIN2 polarity, vesicle trafficking and actin filament architecture. New Phytologist 202, 1184–1196. 24571327 10.1111/nph.12744

[CIT0053] Péret B De Rybel B Casimiro I Benková E Swarup R Laplaze L Beeckman T Bennett MJ 2009 Arabidopsis lateral root development: an emerging story. Trends in Plant Science in 14, 399–408. 10.1016/j.tplants.2009.05.002 19559642

[CIT0054] Pérez-Torres C-A López-Bucio J Cruz-Ramírez A Ibarra-Laclette E Dharmasiri S Estelle M Herrera-Estrella L 2008 Phosphate availability alters lateral root development in *Arabidopsis* by modulating auxin sensitivity via a mechanism involving the TIR1 auxin receptor. Plant Cell 20, 3258–3272. 19106375 10.1105/tpc.108.058719 PMC2630440

[CIT0055] Rasmussen A Mason MG De Cuyper C 2012 Strigolactones suppress adventitious rooting in Arabidopsis and pea. Plant Physiology 158, 1976–1987. 22323776 10.1104/pp.111.187104 PMC3320200

[CIT0056] Rasmussen A Depuydt S Goormachtig S Geelen D 2013a Strigolactones fine-tune the root system. Planta 238, 615–626. 23801297 10.1007/s00425-013-1911-3

[CIT0057] Rasmussen A Heugebaert T Matthys C Van Deun R Boyer F-D Goormachtig S Stevens C Geelen D 2013b A fluorescent alternative to the synthetic strigolactone GR24. Molecular Plant 6, 100–112. 23024210 10.1093/mp/sss110

[CIT0058] Riefler M Novak O Strnad M Schmülling T 2006 *Arabidopsis* cytokinin receptor mutants reveal functions in shoot growth, leaf senescence, seed size, germination, root development, and cytokinin metabolism. Plant Cell 18, 40–54. 16361392 10.1105/tpc.105.037796 PMC1323483

[CIT0059] Ruyter-Spira C Kohlen W Charnikhova T 2011 Physiological effects of the synthetic strigolactone analog GR24 on root system architecture in Arabidopsis: another belowground role for strigolactones? Plant Physiology 155, 721–734. 21119044 10.1104/pp.110.166645 PMC3032462

[CIT0060] Růžička K Ljung K Vanneste S Podhorská R Beeckman T Friml J Benková E 2007 Ethylene regulates root growth through effects on auxin biosynthesis and transport-dependent auxin distribution. Plant Cell 19, 2197–2212. 17630274 10.1105/tpc.107.052126 PMC1955700

[CIT0061] Růžička K Šimášková M Duclercq J Petrášek J Zažímalová E Simon S Friml J Van Montagu MCE Benková E 2009 Cytokinin regulates root meristem activity via modulation of the polar auxin transport. Proceedings of the National Academy of Sciences, USA 106, 4284–4289. 10.1073/pnas.0900060106 PMC2657394 19246387

[CIT0062] Shen H Luong P Huq E 2007 The F-Box protein MAX2 functions as a positive regulator of photomorphogenesis in Arabidopsis. Plant Physiology 145, 1471–1483. 17951458 10.1104/pp.107.107227 PMC2151697

[CIT0063] Shen H Zhu L Bu Q-Y Huq E 2012 MAX2 affects multiple hormones to promote photomorphogenesis. Molecular Plant 5, 750–762. 22466576 10.1093/mp/sss029

[CIT0064] Shinohara N Taylor C Leyser O 2013 Strigolactone can promote or inhibit shoot branching by triggering rapid depletion of the auxin efflux protein PIN1 from the plasma membrane. PLoS Biology 11, e1001474. 23382651 10.1371/journal.pbio.1001474 PMC3558495

[CIT0065] Snowden KC Simkin AJ Janssen BJ Templeton KR Loucas HM Simons JL Karunairetnam S Gleave AP Clark DG Klee HJ 2005 The *Decreased apical dominance1/Petunia hybrida CAROTENOID CLEAVAGE DIOXYGENASE8* gene affects branch production and plays a role in leaf senescence, root growth, and flower development. Plant Cell 17, 746–759. 15705953 10.1105/tpc.104.027714 PMC1069696

[CIT0066] Stirnberg P Ward S Leyser O 2010 Auxin and strigolactones in shoot branching: intimately connected? Biochemical Society Transactions 38, 717–722. 20298249 10.1042/BST0380717

[CIT0067] Sun H Tao J Liu S Huang S Chen S Xie X Yoneyama K Zhang Y Xu G 2014 Strigolactones are involved in phosphate- and nitrate-deficiency-induced root development and auxin transport in rice. Journal of Experimental Botany 65, 6735–6746. 24596173 10.1093/jxb/eru029 PMC4246174

[CIT0068] Tian Q Reed JW 1999 Control of auxin-regulated root development by the *Arabidopsis thaliana SHY2/IAA3* gene. Development 126, 711–721. 9895319 10.1242/dev.126.4.711

[CIT0069] To JPC Haberer G Ferreira FJ Deruère J Mason MG Schaller GE Alonso JM Ecker JR Kieber JJ 2004 Type-A Arabidopsis response regulators are partially redundant negative regulators of cytokinin signaling. Plant Cell 16, 658–671. 14973166 10.1105/tpc.018978 PMC385279

[CIT0070] Tsuchiya Y Vidaurre D Toh S Hanada A Nambara E Kamiya Y Yamaguchi S McCourt P 2010 A small-molecule screen identifies new functions for the plant hormone strigolactone. Nature Chemical Biology 6, 741–749. 10.1038/nchembio.435 20818397

[CIT0071] Umehara M Hanada A Yoshida S 2008 Inhibition of shoot branching by new terpenoid plant hormones. Nature 455, 195–200. 18690207 10.1038/nature07272

[CIT0072] Van Norman JM Zhang J Cazzonelli CI Pogson BJ Harrison PJ Bugg TDH Chan KX Thompson AJ Benfey PN 2014 Periodic root branching in *Arabidopsis* requires synthesis of an uncharacterized carotenoid derivative. Proceedings of the National Academy of Sciences, USA 111, E1300–1309. 10.1073/pnas.1403016111 PMC3977299 24639533

[CIT0073] Vanstraelen M Benková E 2012 Hormonal interactions in the regulation of plant development. Annual Review of Cell and Developmental Biology 28, 463–487. 10.1146/annurev-cellbio-101011-155741 22856461

[CIT0074] Woo HR Chung KM Park J-H Oh SA Ahn T Hong SH Jang SK Nam HG 2001 ORE9, an F-box protein that regulates leaf senescence in Arabidopsis. Plant Cell 13, 1779–1790. 11487692 10.1105/TPC.010061 PMC139127

[CIT0075] Xie X Yoneyama K Yoneyama K 2010 The strigolactone story. Annual Review of Phytopathology 48, 93–117. 10.1146/annurev-phyto-073009-114453 20687831

[CIT0076] Zhang S Li G Fang J 2010 The interactions among *DWARF10*, auxin and cytokinin underlie lateral bud outgrowth in rice. Journal of Integrative Plant Biology 52, 626–638. 20590993 10.1111/j.1744-7909.2010.00960.x

[CIT0077] Zhang W To JPC Cheng C-Y Schaller GE Kieber JJ 2011 Type-A response regulators are required for proper root apical meristem function through post-transcriptional regulation of PIN auxin efflux carriers. Plant Journal 68, 1–10. 21645147 10.1111/j.1365-313X.2011.04668.x

[CIT0078] Zhao L-H Zhou XE Wu Z-S 2013 Crystal structures of two phytohormone signal-transducing α/β hydrolases: karrikin-signaling KAI2 and strigolactone-signaling DWARF14. Cell Research 23, 436–439. 23381136 10.1038/cr.2013.19 PMC3587710

[CIT0079] Zhao Y Christensen SK Fankhauser X Cashman JR Cohen JD Weigel D Chory J 2001 A role for flavin monooxygenase-like enzymes in auxin biosynthesis. Science 291, 306–309. 11209081 10.1126/science.291.5502.306

