# Supplementary material for: Strigolactones spatially influence lateral root development through the cytokinin signaling network
Source: J Exp Bot. 2015 Oct 31;67(1):379–89. doi: 10.1093/jxb/erv478 (PMC4682444; doi:10.1093/jxb/erv478)
Supplement: Supplementary Data [file supp_67_1_379__index.html]

Strigolactones spatially influence lateral root development through the cytokinin signaling network — Strigolactones spatially influence lateral root development through the cytokinin signaling network — Supplementary Data 

# Strigolactones spatially influence lateral root development through the cytokinin signaling network

## Supplementary Data

Data files

- Supplementary Data - Supplementary Data
