# Supplementary material for: Strigolactones spatially influence lateral root development through the cytokinin signaling network
Source: J Exp Bot. 2015 Oct 31;67(1):379–89. doi: 10.1093/jxb/erv478 (PMC4682444; doi:10.1093/jxb/erv478)
Supplement: Supplementary Data [file supp_erv478_Supplementary_figures_S1_S4.pdf]

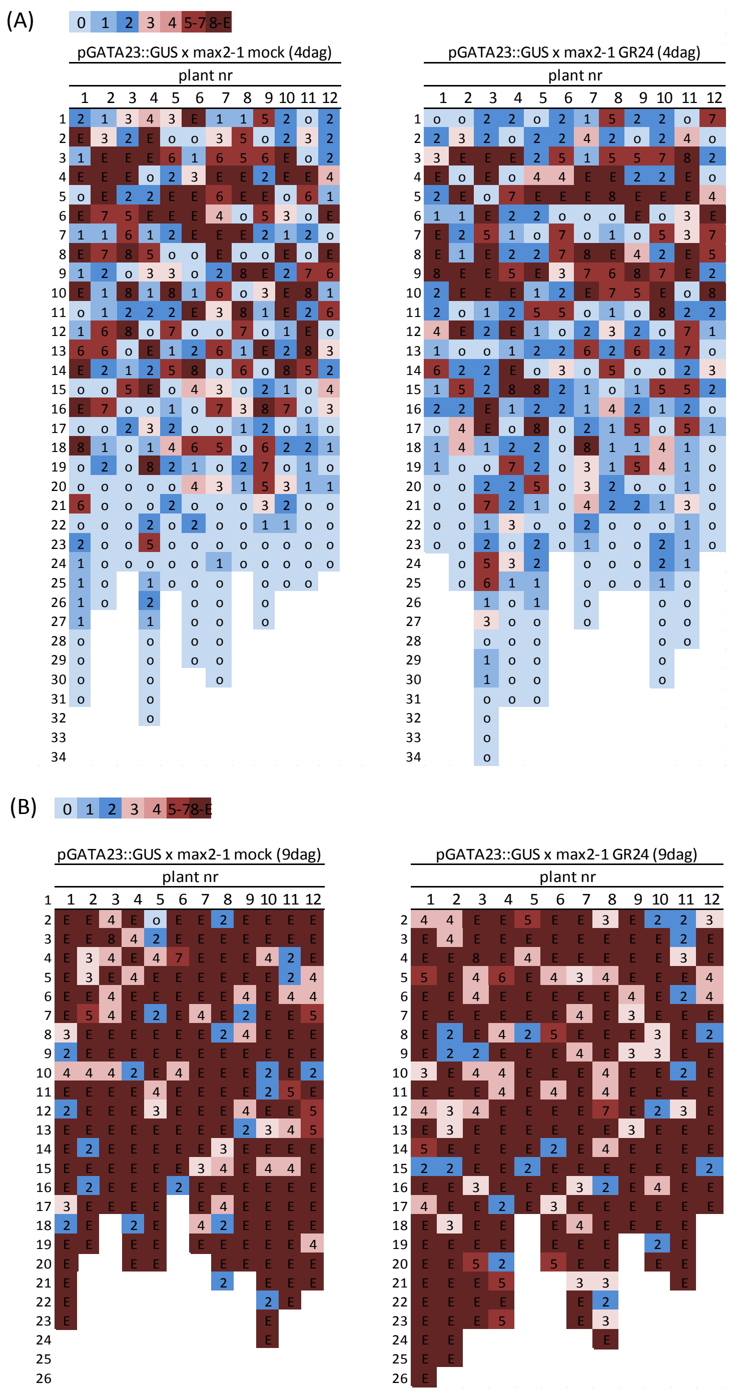

Supplemental Figure S1. Stages of lateral root primordia via GATA23:GUS staining in max2-1 under mock and GR24 treatment at 4 and 9 DAG. Stages of LR primordia via GATA23:GUS staining in *max2-1* under mock (left) and GR24 treatment (right) at 4 DAG (A) and 9 DAG (B). All events, possibly leading to emerged LRs, were scored in individual plants, color-coded, and for each plant, vertically ordered from the closest to the hypocotyl (up) downward to the meristem (down). The root fragments used for analysis were comparable in length.

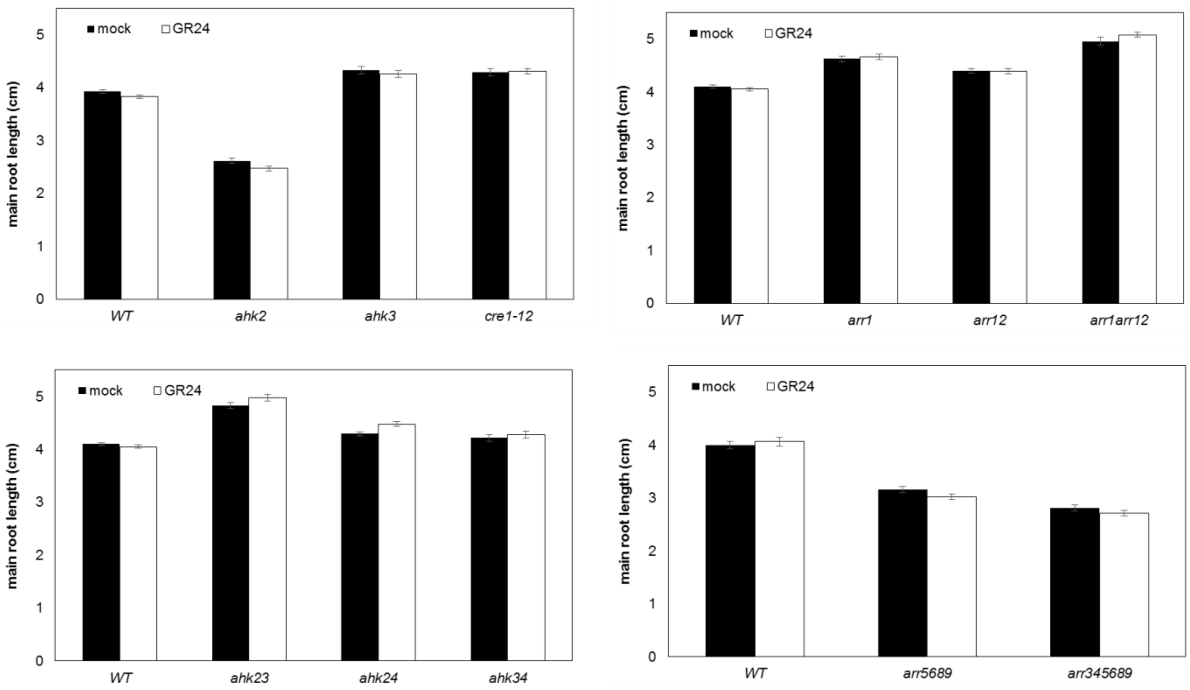

Supplemental Figure S2. Main root lengths of WT and cytokinin receptor and signal transduction mutants under mock and GR24 treatment. Main root lenght of single cytokinin receptor mutants (*ahk2*, *ahk3* and *cre1*), double cytokinin receptor mutants (*ahk2;3*; *ahk2;4* and *ahk3;4*), B-type response regulators (*arr1*, *arr12* and *arr1;12*), and mutants in higher-order A-type response regulators (*arr5;6;8;9* and *arr3;4;5;6;8;9*) upon GR24 treatment. Data presented are means ± SE of three biological repeats (n > 20).

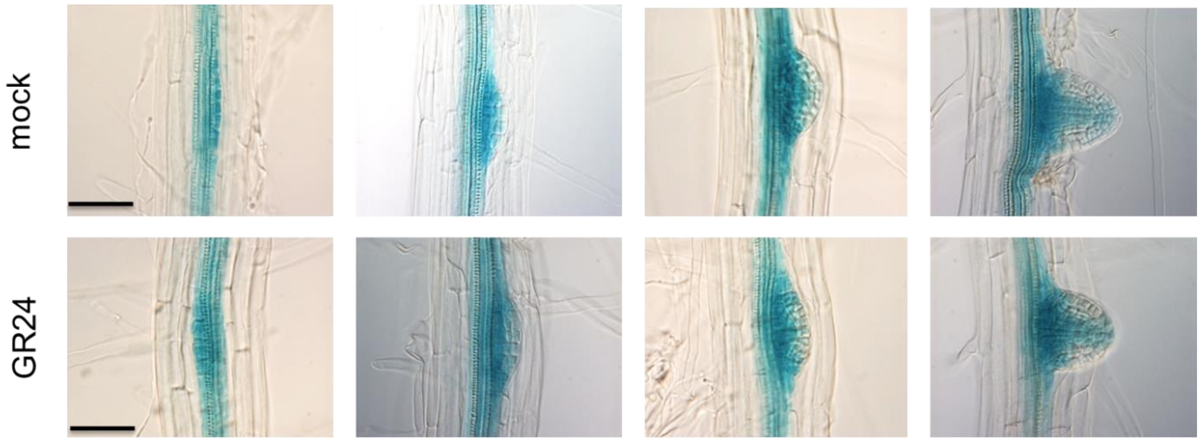

Supplemental Figure S3. p*AHK3-GUS* expression patterns of lateral root primordia at different developmental stages under mock and GR24 treatment. p*AHK3:GUS* expression patterns of lateral root primordia of plants grown with and without *GR24*, 7 days after growth.

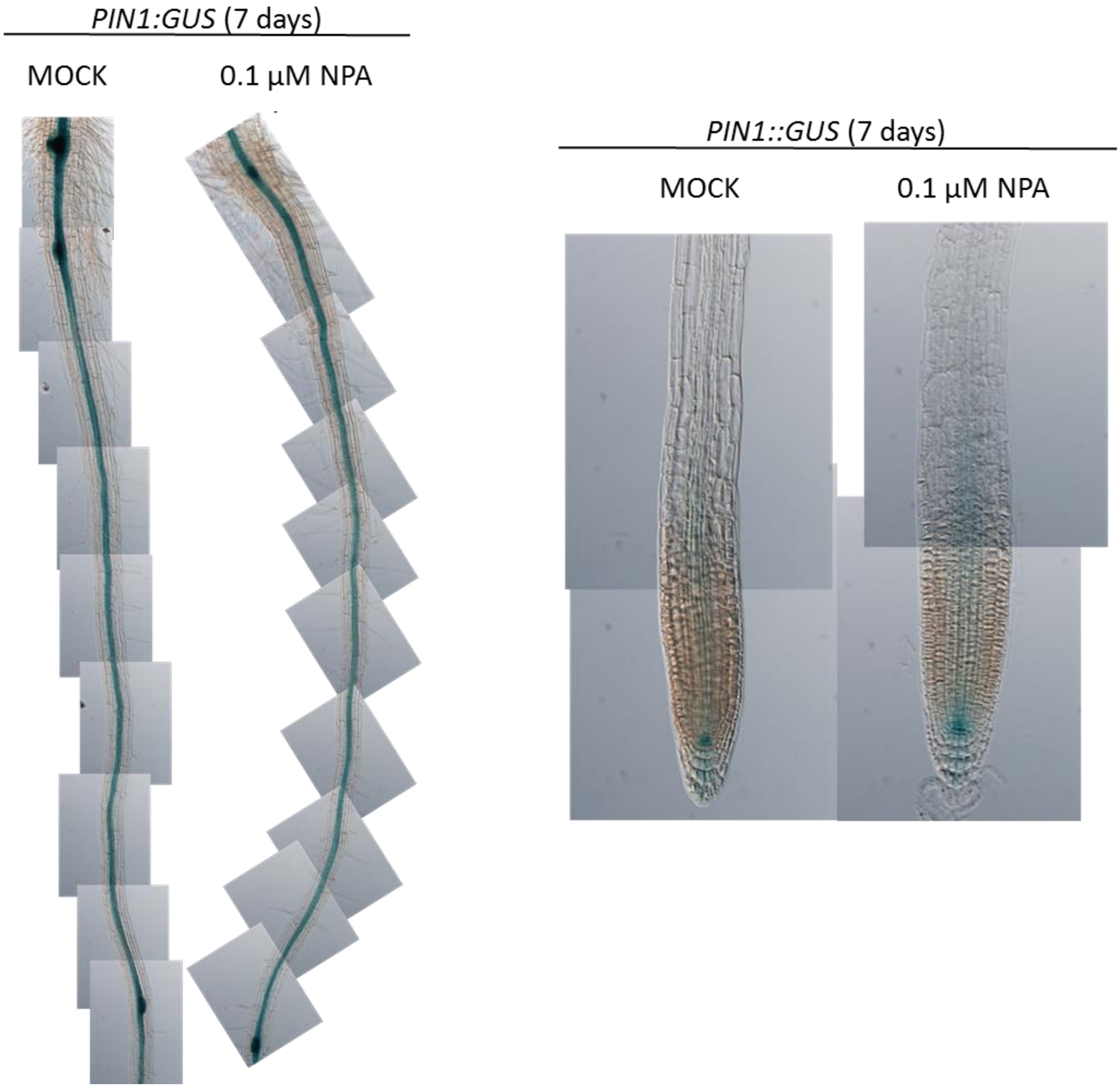

Supplemental Figure S4. *pPIN1:GUS* expression pattern after treatment with 0.1 µM NPA around the root-shoot junction (left) and the root meristem zone (right). p*PIN1:GUS* expression patterns of plants grown with and without *0.1 µM NPA,* 7 days after growth. The region around the root-shoot junction until the first emerged lateral root primordium (left) and the root meristem zone (right) are shown.
